# Exacerbation of autoimmune pulmonary alveolar proteinosis that improved with lone treatment of complicating nontuberculous mycobacterial infection: A case report

**DOI:** 10.1016/j.rmcr.2021.101521

**Published:** 2021-09-27

**Authors:** Shunya Shiohira, Masashi Sakayori, Keiichiro Yoshioka, Hajime Kasai, Ryutaro Hirama, Mitsuhiro Abe, Hiroki Nishimura, Takuji Suzuki

**Affiliations:** a Department of Medicine, School of Medicine, Chiba University, Chiba, 260-8670 Japan; b Department of Respirology, Graduate School of Medicine, Chiba University, Chiba, 260-8670, Japan; c Health Professional Development Center, Chiba University Hospital, Chiba, 260-8670, Japan; d Department of Respiratory Medicine, National Hospital Organization Chiba Medical Center, Chiba, 260-0042, Japan

**Keywords:** Antibiotics, Exacerbation, *Mycobacterium avium* complex, Pulmonary alveolar proteinosis

## Abstract

Herein, we present the case of a 63-year-old man with autoimmune pulmonary alveolar proteinosis (APAP) complicated by *Mycobacterium avium* complex (MAC) infection. APAP was diagnosed based on serum anti-granulocyte-macrophage colony-stimulating factor antibody, bronchoalveolar lavage fluid (BALF) findings, and transbronchial lung biopsy. Nodular shadows with cavities were visible on chest CT images, and *Mycobacterium intracellulare* was identified by BALF culture. Rifampicin, ethambutol, and clarithromycin were administered, and 4 months later, the nodular shadows of MAC had disappeared, and APAP was remarkably improved. Thus, in cases of APAP exacerbation complicated with infections, such as MAC, control of the infections may improve APAP.

## 1 Introduction

Pulmonary alveolar proteinosis (PAP) is a rare syndrome characterized by the accumulation of surfactants in alveolar macrophages and alveoli, possibly resulting in hypoxemic respiratory failure [1]. PAP is classified into three groups: primary PAP, secondary PAP, and congenital PAP [2]. Autoimmune PAP (APAP) is the primary type and accounts for over 90% of all PAP [2]. While mild forms of PAP simply require monitoring, treatments, such as whole-lung lavage (WLL), are required in severe and/or disabling cases of APAP [2].

PAP can be complicated by mycobacterial infections, and tuberculosis is the most common [3]. However, complications of PAP due to *Mycobacterium avium* complex (MAC) infection are relatively rare. Furthermore, there is no established strategy for treating exacerbations of APAP associated with infection, including MAC.

Herein, we present a case of improved APAP when treated only with antimicrobials against coexisting MAC infection.

## 2 Case presentation

**Fig. 1:**
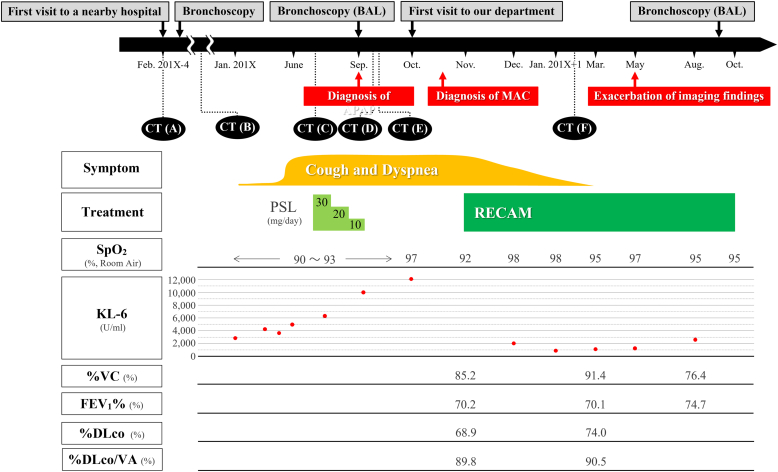
The clinical course of the patient. APAP, autoimmune pulmonary alveolar proteinosis; CT, computed tomography; DLco, carbon monoxide diffusing capacity; FEV1%, forced expiratory volume 1.0 (sec) percent; MAC, *Mycobacterium avium* complex; PSL, prednisolone; RECAM, treatment with rifampicin, ethambutol, and clarithromycin; VA, alveolar volume; VC, percent vital capacity.

**Fig. 2:**
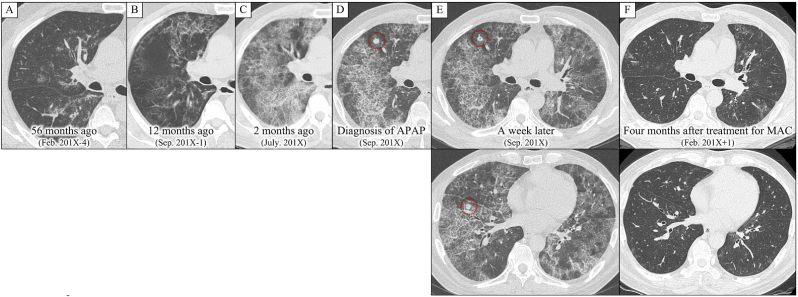
Chest computed tomography (CT) on the first visit to a nearby hospital demonstrated patchy bilateral ground-glass attenuation (GGA) (A). The patient was followed-up on suspicion of interstitial pneumonia, and the GGA in both lungs worsened gradually (B-D). Chest CT at the time of diagnosis of autoimmune pulmonary alveolar proteinosis demonstrated nodules in both lungs (dotted area) (D), and cavities were visible within the nodules after 1 week (dotted area) (E). After a 6-month treatment with rifampicin, ethambutol, and clarithromycin, the GGA and nodules were improved remarkably (F). APAP, autoimmune pulmonary alveolar proteinosis; MAC, *Mycobacterium avium* complex.

**Fig. 3:**
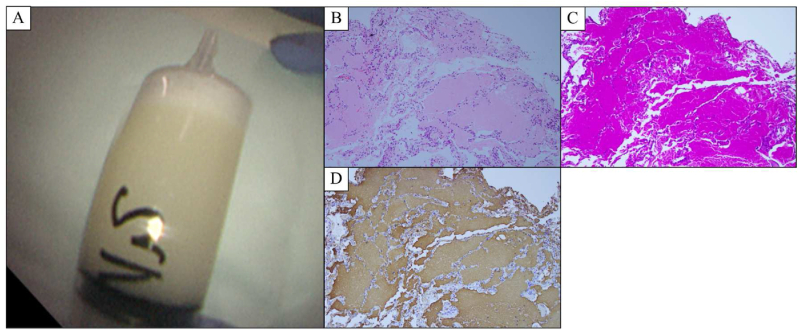
The bronchoalveolar lavage fluid had a milky appearance (A). Transbronchial lung biopsy specimen demonstrated peripheral air spaces filled with granular eosinophilic amorphous material (B, hematoxylin and eosin staining, × 100). The material was positive in periodic acid-Schiff staining (C, × 100) and hydrophilic surfactant proteins A staining (D, × 100).

A 63-year-old man was referred to our department for the treatment of exacerbation of APAP (October 201X). Four years and eight months ago, he was admitted to a nearby hospital. In a health check-up, an abnormal shadow was seen on a chest radiograph (Fig. 1). Chest computed tomography (CT) showed bilateral ground-glass attenuation (GGA) (Fig. 2A), and the patient was followed-up on suspicion of interstitial pneumonia. The bilateral GGA gradually worsened (Fig. 2B). Four months before his first visit to our hospital, he developed cough and hypoxemia. Corticosteroids (prednisolone) at 30 mg per day were then administered. However, because his symptoms and imaging findings did not improve (Fig. 2C), corticosteroids treatment was tapered and discontinued after 2.5 months. One month before his first visit to our department, APAP was diagnosed based on a positive serum anti-granulocyte-macrophage colony-stimulating factor (GM-CSF) antibody, bronchoalveolar lavage fluid (BALF) findings, and transbronchial lung biopsy results (Fig. 3). Chest CT performed at the time of diagnosis revealed multiple new nodular shadows and diffuse GGA with a crazy-paving appearance (Fig. 2D). These nodular shadows were accompanied by cavities on CT images taken after 1 week (Fig. 2E).

At the first visit to our department, his SpO_2_ was 97% in ambient air. In the past, he smoked 40–60 cigarettes daily and was still smoking 4–5 cigarettes daily at that time. Pulmonary function tests (PFTs) at the time of the first visit revealed vital capacity of 85.2%, forced expiratory volume 1.0 (sec) of 70.2%, carbon monoxide diffusing capacity (DLco) of 68.9%, and DLco per alveolar volume of 89.8%. A chest radiograph demonstrated diffuse GGA in both lung fields. Blood examination revealed a lactate dehydrogenase level of 295 U/L and a KL-6 level of 12,110 U/mL, with no elevated levels of inflammatory markers (Table 1). Arterial blood gas analysis showed A-aDO_2_ widening (33 mmHg). Furthermore, although acid-fast smear and polymerase chain reaction results for *Mycobacteriumu avium complex* were negative, *Mycobacterium intracellulare* was identified after 3 weeks of BALF culturing at the nearby hospital. Based on the appearance of nodular shadows with cavities on chest CT and the detection of *M. intracellulare* from BALF, APAP complicated by MAC infection was diagnosed.

**Table 1:** Laboratory data on admission.

Complete blood count			Blood chemistry			Immunology		
White blood cell count	6800	/μg	Aspartate aminotransferase	18	U/L	C-reactive protein	0.1	mg/dL
Neutrophil	59.0	%	Alanine aminotransferase	11	U/L	Carcinoembryonic antigen	10.0	ng/mL
Eosinophil	2.3	%	Lactate dehydrogenase	295	U/L	Krebs von den Lungen-6	12110	ng/mL
Monocyte	8.1	%	Alkaline phosphatase	321	U/L	Anti-GM-CSF Antibody	57.2	pg/mL
Lymphocyte	30.0	%	γ-glutamyl transferase	21	U/L	Soluble Interleukin-2 Receptor	514	U/ml
Red blood cell count	4.63x10^4^	/μL	Total bilirubin	0.7	mg/dL			
Hemoglobin	14.5	g/dL	Total protein	6.4	g/dL			
Hematocrit	43.4	%	Albumin	3.6	g/dL			
Platelet count	23.1 × 10^4^	/μL	Urea nitrogen	15.0	mg/dL			
			Creatinine	0.5	mg/dL			
			Sodium	139.0	mmol/L			
			Potassium	4.2	mmol/L			
			Chlorine	107.0	mmol/L			

GM-CSF: granulocyte-macrophage colony-stimulating factor.

Because of the risk of MAC dissemination during WLL, the procedure was deferred, and antimicrobial treatment for MAC was initiated 1 month after his first visit to our department. After 4 months of treatment with rifampicin (450 mg/day), ethambutol (750 mg/day), and clarithromycin (800 mg/day) (RECAM), not only had the SpO_2_ increased but the imaging findings also remarkably improved (Fig. 2F). In addition, PFTs after 5 months of treatment with RECAM showed slight improvements (Fig. 1). However, 7 months after the administration of RECAM, the GGA worsened again. Bronchoalveolar lavage performed after 11 months of treatment with RECAM showed absence of *M. intracellulare*. Therefore, an exacerbation of APAP was suspected, and RECAM was discontinued after approximately 12 months of treatment. After 11 months of discontinuation, the patient underwent WLL for APAP exacerbation, and no recurrence of MAC infection was noted.

## 3 Discussion

The present case demonstrates two notable clinical findings. First, in rare cases, APAP can be complicated by MAC infection. Second, in the case of APAP complicated by MAC infection, control of infection by treatment for MAC may also contribute to the improvement of APAP.

It has been reported that 5–13% of patients with APAP develop secondary infections [2]. In a literature review by Punatar et al. [3], among 75 cases of PAP with opportunistic infections, 28 (37%) were associated with mycobacterial infections. Of these 28 cases, 21 (75%) were caused by *Mycobacterium tuberculosis*, and MAC was seen in only three cases (14.3%). Furthermore, these opportunistic infections may precede or follow the diagnosis of APAP. On the other hand, it has been reported that the detection rate of MAC in BALF is higher in PAP [4]. Therefore, it is necessary to make a careful judgment whether MAC infection is established. In our case, in addition to the detection of MAC in BALF, the appearance of a nodular shadow with cavities on CT and the response to RECAM were consistent with pulmonary MAC infection. Additionally, Akasaka et al. reported that among 31 cases of APAP with corticosteroids therapy, 23 (74.1%) had worsened APAP, and six had new complications of infection, suggesting that corticosteroids might worsen APAP and increase the risk for infections [5]. In our case, the possibility that corticosteroids were involved with the emergence of MAC infection cannot be excluded. Although it is unclear whether MAC infection was caused by corticosteroid-induced immunosuppression or by corticosteroid-induced manifestation of pre-existing MAC, it was highly likely that the corticosteroids had a negative effect on MAC infection. APAP may be complicated by opportunistic infections, including MAC, and it is important to evaluate for *Mycobacterium* infections in BALF and image findings.

Infections including MAC may be associated with exacerbation of APAP, and treatment for the infections may also contribute to the improvement of APAP. In a case of PAP with pulmonary tuberculosis [6] and another with nocardiosis [7], treatment of the infections resulted in the improvement of PAP. However, there is a case report that WLL improved PAP associated with pulmonary tuberculosis, although tuberculosis itself worsened [8]. Therefore, the possibility of the spread of infection by WLL should not be ignored. Tachibana et al. [9] reported a case of PAP complicated by MAC infection that was improved using WLL in the right lung and antibiotic therapy for MAC in the left lung. In that case, chest imaging revealed improvement in not only the infectious lesions but also PAP in the left lung. In our case, 3 months after the administration of RECAM, not only the nodular shadow caused by MAC disappeared but also the imaging evidence of APAP improved remarkably (Fig. 2F). Additionally, exacerbation of PAP with corticosteroids treatment itself has been reported [5]. In our case, corticosteroids were administered for gradually worsening APAP imaging findings and hypoxemia, which may have caused further worsening of APAP. There was a possibility that APAP may have improved with the discontinuation of corticosteroids; however, the imaging findings after MAC treatment were even better than before the introduction of corticosteroids. This suggested that MAC treatment may be most likely to contribute to the improvement of APAP exacerbation. The infection may have impaired GM-CSF production, affected macrophages and neutrophils, and exacerbated APAP. Moreover, it has been reported [9] that treatment of the infection may restore alveolar macrophage function and the ability to remove surfactants. At that point, it is most likely that control of infection by RECAM contributed to the improvement of APAP. Four months after RECAM was administered, the chest CT images worsened, and WLL was then performed. As MAC was already undetectable in the BALF of the WLL, APAP itself could have been worsening.

There are reports of the spontaneous remission of PAP [10,11]. This possibility cannot be completely excluded in the present case. However, the dramatic improvement in APAP-induced GGA coincided with the time course and response of the MAC-induced nodular shadow to treatment with RECAM. Therefore, treatment of concomitant MAC could be effective in resolving an exacerbation of APAP. Although treatment of infections cannot serve as definitive treatment for APAP, treatment of complicating infections prior to WLL can reduce the severity of APAP in addition to reducing the risk of dissemination of pathogens throughout the lungs. Because it is unclear how long the improvement in APAP can be sustained by treatment of the infection, accumulation of further data is required.

## 4 Conclusion

APAP can be complicated opportunistic infections, including MAC and exacerbated by the infections. In cases of exacerbation of APAP, it may be essential to search for infectious etiologies, including MAC. Control of infection prior to WLL may be effective for the improvement of APAP.

## Funding

This case report did not receive any specific grant from funding agencies in the public, commercial, or not-for-profit sectors.

## Declaration of competing interest

None.
